# Systemic Metabolic Alterations Induced by Etodolac in Healthy Individuals

**DOI:** 10.3390/ph18081155

**Published:** 2025-08-04

**Authors:** Rajaa Sebaa, Reem H. AlMalki, Hatouf Sukkarieh, Lina A. Dahabiyeh, Maha Al Mogren, Tawfiq Arafat, Ahmed H. Mujamammi, Essa M. Sabi, Anas M. Abdel Rahman

**Affiliations:** 1 Department of Medical Laboratories, College of Applied Medical Sciences, Shaqra University, Shaqra 11961, Saudi Arabia; r.sebaa@su.edu.sa; 2 Metabolomics Section, Precision Medicine Laboratory Department, Genomics Medicine Center of Excellence, King Faisal Specialist Hospital and Research Center, Riyadh 11211, Saudi Arabia; rgalmalki@kfshrc.edu.sa (R.H.A.); mmogren@kfshrc.edu.sa (M.A.M.); 3 Department of Pharmacology, College of Medicine, Alfaisal University, Riyadh 11533, Saudi Arabia; hsukkarieh@alfaisal.edu; 4 Department of Pharmaceutical Sciences, School of Pharmacy, The University of Jordan, Amman 11972, Jordan; dahabiyeh@ju.edu.jo; 5 Jordan Center for Pharmaceutical Research (JCPR), Amman 11118, Jordan; tawfiqarafat@yahoo.com; 6 Clinical Biochemistry Unit, Pathology Department, College of Medicine, King Saud University, Riyadh 11461, Saudi Arabia; amujammai@ksu.edu.sa (A.H.M.); esabi@ksu.edu.sa (E.M.S.); 7 Department of Biochemistry and Molecular Medicine, College of Medicine, Alfaisal University, Riyadh 11533, Saudi Arabia

**Keywords:** LC/MS-based untargeted metabolomics, Etodolac, metabolic alterations, metabolic profiling, metabolic pathways

## Abstract

**Background/Objective:** Pharmacological interventions often exert systemic effects beyond their primary targets, underscoring the need for a comprehensive evaluation of their metabolic impact. Etodolac is a nonsteroidal anti-inflammatory drug (NSAID) that alleviates pain, fever, and inflammation by inhibiting cyclooxygenase-2 (COX-2), thereby reducing prostaglandin synthesis. While its pharmacological effects are well known, the broader metabolic impact and potential mechanisms underlying improved clinical outcomes remain underexplored. Untargeted metabolomics, which profiles the metabolome without prior selection, is an emerging tool in clinical pharmacology for elucidating drug-induced metabolic changes. In this study, untargeted metabolomics was applied to investigate metabolic changes following a single oral dose of etodolac in healthy male volunteers. By analyzing serial blood samples over time, we identified endogenous metabolites whose concentrations were positively or inversely associated with the drug’s plasma levels. This approach provides a window into both therapeutic pathways and potential off-target effects, offering a promising strategy for early-stage drug evaluation and multi-target discovery using minimal human exposure. **Methods:** Thirty healthy participants received a 400 mg dose of Etodolac. Plasma samples were collected at five time points: pre-dose, before Cmax, at Cmax, after Cmax, and 36 h post-dose (*n* = 150). Samples underwent LC/MS-based untargeted metabolomics profiling and pharmacokinetic analysis. A total of 997 metabolites were significantly dysregulated between the pre-dose and Cmax time points, with 875 upregulated and 122 downregulated. Among these, 80 human endogenous metabolites were identified as being influenced by Etodolac. **Results:** A total of 17 metabolites exhibited time-dependent changes closely aligned with Etodolac’s pharmacokinetic profile, while 27 displayed inverse trends. **Conclusions:** Etodolac influences various metabolic pathways, including arachidonic acid metabolism, sphingolipid metabolism, and the biosynthesis of unsaturated fatty acids. These selective metabolic alterations complement its COX-2 inhibition and may contribute to its anti-inflammatory effects. This study provides new insights into Etodolac’s metabolic impact under healthy conditions and may inform future therapeutic strategies targeting inflammation.

## 1. Introduction

Etodolac is a nonsteroidal anti-inflammatory drug (NSAID) that has analgesic characteristics. In 1991, Etodolac was approved by the Food and Drug Administration (FDA) as an analgesic and anti-inflammatory medication [1,2,3]. Etodolac is considered a preferentially selective cyclo-oxygenase-2 (COX-2) inhibitor. It offers fewer GI side effects than non-selective NSAIDs, but not to the extent of fully selective COX-2 inhibitors [4,5,6]. Inhibition of COX-2 results in decreased levels of corresponding prostaglandins (PGs), which are contributing factors in inflammation and pain [7,8,9].

Pharmacokinetic studies have shown that Etodolac is rapidly and well absorbed after oral administration. It binds to plasma proteins with high affinity. Maximal plasma concentration is achieved within 1 to 2 h in healthy individuals [10]. Etodolac is metabolized hepatically through cytochrome P450 enzymes, producing three inactive metabolites, including a glucuronide metabolite, a hydroxylated metabolite, and glucuronidated hydroxylated metabolites. These metabolites are all eliminated renally. The elimination half-life of Etodolac is approximately 6–8 h [10,11,12].

Clinically, Etodolac is used to relieve a range of symptoms, including fever, pain, and inflammation associated with conditions such as rheumatoid arthritis, osteoarthritis, pyrexia, gout, migraines, and musculoskeletal disorders [1,2].

Untargeted metabolomics is considered a gold standard in pharmacology for gaining deeper insights into the metabolic effects of drugs in both health and disease. This approach enables the comprehensive detection of a broad spectrum of metabolic alterations within a biological system in response to drug exposure. Untargeted metabolomics can also predict drug-related toxicity doses and detect drug-linked therapeutic potentials [13,14,15,16].

Despite its long-standing clinical use, the systemic metabolic effects of Etodolac have not been comprehensively characterized under healthy physiological conditions. While several NSAIDs have been studied using metabolomics to explore off-target effects and systemic toxicity, similar investigations specifically targeting Etodolac are lacking [17,18,19]. This study addresses that gap by using an untargeted metabolomics approach to uncover potential mechanistic insights, safety markers, and future opportunities for personalized anti-inflammatory therapy [17].

The ability to understand the systemic effects of a pharmaceutical compound following minimal exposure is a growing priority in modern drug development. Traditionally, such insights have required longer-term studies or disease-specific cohorts, increasing cost and complexity while potentially obscuring early metabolic signals [20]. In this study, we explored a novel application of untargeted metabolomics to profile the dynamic metabolic responses to a single oral dose of etodolac in well-characterized healthy male volunteers. By capturing temporal metabolomic shifts that align with the drug’s pharmacokinetics, we identified endogenous metabolites exhibiting dose-correlated behavior, either in parallel with or in opposition to the drug’s plasma concentration. These metabolites were further investigated for their relevance to known therapeutic pathways and possible side effect mechanisms. This strategy highlights the utility of single-dose studies not only in assessing pharmacodynamic responses but also in mapping broader biological effects, aiding in the identification of multi-therapeutic targets and early indicators of drug safety or repurposing potential.

## 2. Results

### 2.1. Demographics and Clinical Characteristics of Participants

All clinical assessments were performed prior to Etodolac administration and blood sample collection. The study enrolled healthy male volunteers, confirmed through demographic data and clinical evaluation. Participants ranged in age from 19 to 41 years (mean age: 25 ± 6.8), with body mass index (BMI) values ranging from 18.8 and 29 kg/m^2^ (mean BMI: 23.8 ± 3.29). Biochemical parameters, including fasting blood sugar, urea, creatinine, sodium, potassium, SGOT, SGPT, ALP, and total bilirubin, were all within normal limites. Hematological parameters, such as red blood cell count, hemoglobin, hematocrit, MCV, MCH, MCHC, and white blood cell count, were also within normal ranges. Furthermore, immunological assessments and urine analysis showed no abnormalities (Table 1 and Tables S1–S6).

### 2.2. Pharmacokinetics of Etodolac in All Participants

**Figure 1:**
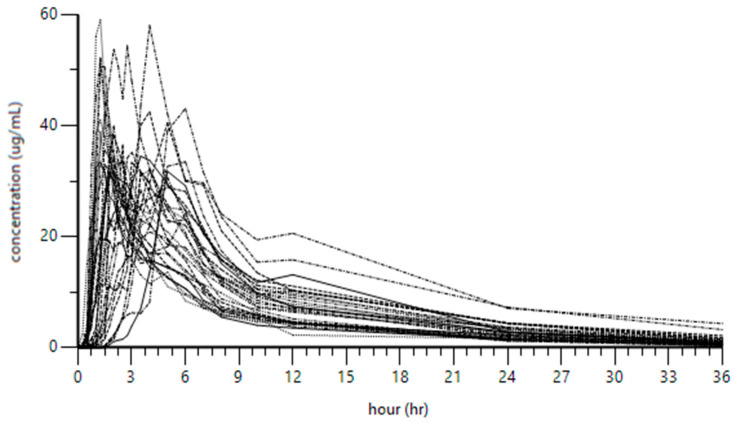
The pharmacokinetics of Etodolac in all healthy participants.

For pharmacokinetic analysis, blood sampling began with a baseline pre-dose sample from all participants, referred to as the pre-Etodolac dose sample. After oral administration of a 400 mg dose of Etodolac, four additional blood samples were collected at defined time points: prior to reaching Cmax, at Cmax, after Cmax, and 36 h post-dose. Etodolac concentrations were measured in all five samples to construct the drug’s pharmacokinetic profile across participants, as illustrated in Figure 1.

The results indicated that all 90% confidence intervals, as determined by ANOVA, fell within the predefined acceptable ranges (Cmax: 91.18–105.29; AUC0-t: 90.96–98.23)

### 2.3. Metabolic Alterations Associated with Etodolac Administration

A total of 21,813 and 10,524 mass ion features were detected in the positive and negative ionization modes, respectively. After applying a frequency filter with an 80% occurrence threshold across all samples, 23,653 ion features remained. These features were statistically analyzed across the five time points (pre-dose, before Cmax, at Cmax, after Cmax, and 36 h post-dose) using one-way ANOVA (Tukey’s post hoc, FDR *p* < 0.05). This analysis identified 2488 mass ions that were significantly dysregulated across the different time points.

**Figure 2:**
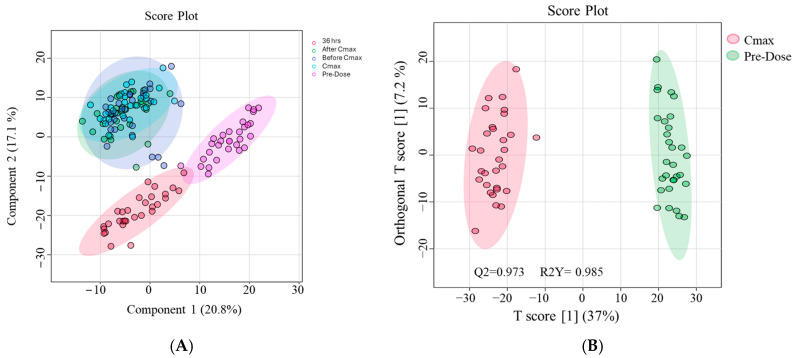
(**A**) Partial least squares discriminant analysis (PLS-DA) based on 2488 mass ions showing semi-separation between the groups. (**B**) The OPLS-DA model based on 2488 masses showing evident separation between Cmax and pre-dose scores. The robustness of the created models was evaluated by the fitness of the models (R^2^Y = 0.985) and their predictive ability (Q^2^ = 0.973) values in a larger dataset (*n* = 100).

A partial least squares discriminant analysis (PLS-DA) score plot based on 2488 mass ions from the metabolic profiles of the 30 healthy subjects across the five time points revealed partial separation among the time points (Figure 2A). Further analysis using orthogonal projections to latent structures discriminant analysis (OPLS-DA) comparing the pre-Etodolac dose and Cmax time points showed clear separation between the two, with model performance scores of Q^2^ = 0.973 and R^2^Y = 0.985 (Figure 2B). To check if our models were reliable and not overfitted, we used seven-fold cross-validation and also performed 200 permutation tests in MetaboAnalyst. The results showed that the high R^2^Y and Q^2^ values in both PLS-DA and OPLS-DA were statistically meaningful (*p* < 0.01), and the models were stable.

**Figure 3:**
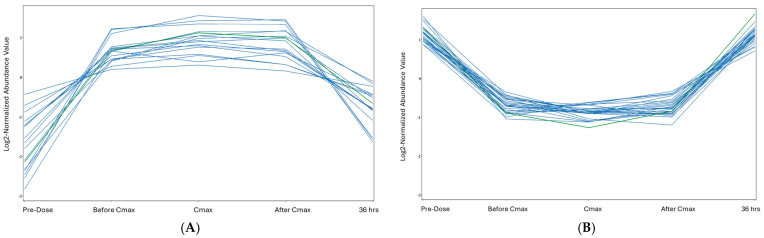
The metabolic profiles associated with Etodolac. (**A**) Seventeen metabolites exhibited temporal patterns that closely mirrored the pharmacokinetic curve of Etodolac. These metabolites were found to be involved in key pathways such as arachidonic acid metabolism (e.g., PIP derivatives), fatty acid metabolism (e.g., palmitoyl-CoA), and amino acid metabolism (e.g., phenylalanine, glutamylalanine), suggesting coordinated or mechanistically linked biological responses. The green curve is the Etodolac reference curve, and the blue curves are for metabolites that follow a similar trend. (**B**) Twenty-seven metabolites displayed an inverse trend relative to Etodolac plasma levels, like prostaglandin biosynthesis (e.g., PGE2, PGF1α), sphingolipid metabolism (e.g., GM1, GlcCer), and phospholipid remodeling (e.g., lysoPA, CDP-DG), indicating potential regulatory or compensatory processes following Etodolac administration. The green curve is the inverse reference of Etodolac (MG(0:0/TXB2/0:0)), objectively selected, while the blue curves represent metabolites following this inverse pattern.

A fold change cut-off of 2 was applied to compare the Cmax and pre-dose time points, identifying 997 significantly dysregulated mass ions (875 upregulated and 122 downregulated). Of these, 564 mass ions were annotated using databases. After excluding exogenous molecules (i.e., drugs, drug metabolites, environmental exposures, etc.), 80 annotated metabolites were identified as human endogenous metabolites. These metabolites showed two distinct patterns relative to etodolac administration, as summarized in Table 2 and Table S7. Out of the 80 metabolites, 17 had a similar pattern to Etodolac (Figure 3A, Table S8). At the same time, the levels of 27 metabolites were opposite to the level pattern of Etodolac, as presented in (Figure 3B and Table S9). Notably, metabolic profiling was conducted across all five time points to assess changes in metabolite levels over time.

**Figure 4:**
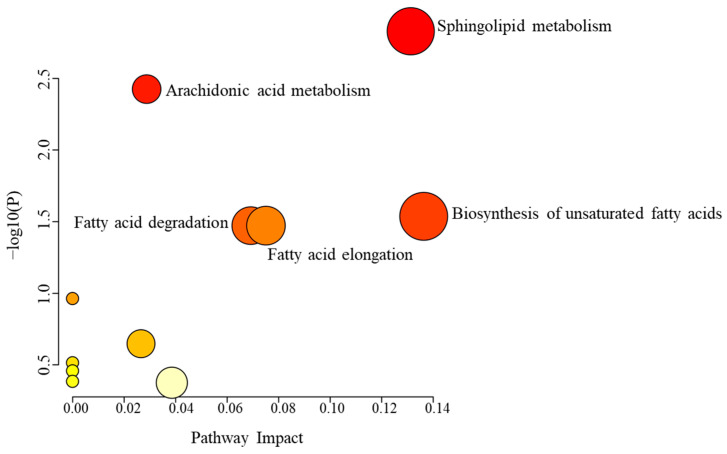
Pathway analysis of 80 significantly dysregulated metabolites affected by Etodolac dose between pre-dose and Cmax samples. Circle color intensity (white to red) reflects increasing statistical significance, and circle diameter varies with the pathway impact.

Pathway analysis revealed that the 80 dysregulated metabolites affected by Etodolac were involved in five key metabolic pathways: sphingolipid metabolism, arachidonic acid metabolism, biosynthesis of unsaturated fatty acids, fatty acid degradation, and fatty acid elongation, as shown in Figure 4.

## 3. Discussion

Etodolac is well recognized for its therapeutic benefits in managing symptoms associated with various types of arthritis and other pain-related conditions, primarily through the reduction of inflammation, swelling, stiffness, and joint pain. Etodolac distinguishes itself from many other NSAIDs by its preferential selectivity; i.e., it may offer fewer GI side effects than non-selective NSAIDs like ibuprofen [21,22]. The majority of published studies on Etodolac have focused mainly on its therapeutic uses and mechanistic actions under pathological conditions [22,23,24,25], with limited exploration of its metabolic effects.

Our study sheds light on the systemic metabolic effects of Etodolac administration under healthy conditions, an area that has not been explored. Our analysis indicates that etodolac administration causes noticeable systemic metabolic alterations, as observed in selective metabolites and metabolic pathways. Moreover, certain altered metabolites exhibit a similar trend in Etodolac-related pharmacokinetics, while other metabolites behave oppositely. Notably, our study not only highlights the impact of Etodolac on its well-known target pathway (arachidonic metabolism), but also uncovers its broader influence on additional metabolic pathways, as discussed below.

### 3.1. Characterizing Systemic Metabolomic Changes Induced by Etodolac in Healthy Individuals

#### 3.1.1. Metabolites Exhibiting Parallel or Divergent Kinetics Relative to Etodolac Pharmacokinetics

In the field of pharmacokinetics, an administered drug undergoes four key processes: absorption, distribution, metabolism, and elimination. Throughout these phases, the body responds to the drug over the entire exposure period, causing various changes [10,15,26]. In this study, we aimed to investigate the metabolic changes of the body occurring in healthy participants during exposure to Etodolac. Thus, we examined whether the systemic metabolic profiles were altered in response to Etodolac administration from the pre-dose time point to 36 h post-dose.

Interestingly, several metabolites exhibited temporal profiles that closely mirrored the pharmacokinetic curve of Etodolac, following a similar directional trend across the five time points (Figure 3) This suggests a coordinated metabolite response potentially linked to Etodolac’s mechanism of action.

In contrast, a distinct group of metabolites showed an inverse trend, indicating opposing metabolic alterations that may reflect compensatory or regulatory processes triggered by drug administration.

Among the metabolites with pharmacokinetic-like curves were phosphoinositide phosphate (PIP), amino acids such as phenylalanine and glutamylalanine, and fatty acids such as Palmitoyl-CoA.

Phosphoinositide phosphates (PIPs), particularly PI(4,5)P_2_, are lipid signaling molecules. PI(4,5)P2 is crucial for cellular functioning, regulating processes like vesicular transport, membrane dynamics, actin cytoskeleton remodeling, and immune cell signaling. Disruptions in PI(4,5)P2 homeostasis can lead to various diseases, including neurological disorders, metabolic issues, and cancer. PIPs are upstream regulators of inflammatory responses and have been implicated in T-cell activation, NF-κB modulation, and cytokine signaling [27,28,29]. They are also known to participate in key processes within the nervous system, including cell signaling, membrane trafficking, and synaptic function [30,31]. PIP2 plays a crucial role in pain management by regulating ion channels and acting as a convergence point for multiple receptors and signaling pathways that sustain chronic pain. Reducing PIP2 levels in neurons has been shown to decrease pronociceptive signaling, suggesting a potential novel approach for pain treatment. However, current first-line therapies, such as NSAIDs, acetaminophen, and opioids, often provide only partial relief and have harmful side effects, highlighting the need for new therapeutic targets that address the complexities of nociceptive signaling [32].

Etodolac administration led to an elevation in palmitoyl-CoA levels, a metabolically active form of palmitic acid and a central intermediate in fatty acid metabolism. This change mirrored Etodolac’s pharmacokinetic profile and suggests a potential metabolic role in modulating inflammation [32]. A study showed that palmitic acid can exhibit diverse pharmacological effects, including mediating hypothalamic insulin resistance and promoting apoptotic activities. While palmitoyl-CoA has been associated with pro-inflammatory states in conditions like metabolic syndrome and diabetes, its increase in this study may indicate a compensatory lipid remodeling response or altered energy metabolism triggered by Etodolac [33]. Some NSAIDs, including Etodolac, are known to influence fatty acid metabolism via PPARα signaling, which may further support this mechanism [34]. Additionally, the elevation of palmitoyl-CoA may contribute to anti-inflammatory effects by engaging stress response pathways, such as the unfolded protein response (UPR), which has been linked to protective effects in inflammatory diseases like osteoarthritis. These findings point to a potentially dual role of palmitoyl-CoA in inflammation—pro- or anti-inflammatory depending on context—highlighting the need for further investigation in pathological states to better understand its contribution to Etodolac’s therapeutic effects [35]. Previous research has shown that palmitic acid can cause the unfolded protein response (UPR). The unfolded protein response (UPR) is activated as a cellular stress response mechanism when misfolded or unfolded proteins accumulate in the endoplasmic reticulum (ER). This response was demonstrated in one study when genes encoding enzymes involved in phospholipid remodeling, such as lysophosphatidylcholine acyltransferase-3 and stearoyl-CoA desaturase-1, were mutated. These enzymes are crucial for maintaining the membrane phospholipid composition and regulating energy metabolism. In parallel, another study revealed that the enhancement of the mitochondrial unfolded protein response (UPRmt) ameliorates osteoarthritis progression, suggesting that the UPRmt exerts a protective effect against osteoarthritis [36]. The potential roles of palmitoyl-CoA through the UPR warrant further investigation. This is a preliminary speculation that requires further experimental validation.

Furthermore, our findings revealed similarities between certain amino acids and Etodolac’s pharmacokinetics, such as phenylalanine and glutamylalanine. Supporting our results, one study showed that glutamine and alanine supplementation helped alleviate muscle fatigue and pain [37]. Conversely, other research showed that phenylalanine metabolism plays a significant role in knee pain [38].

Notably, phenylalanine has been proposed as a potential plasma marker for a higher risk of bilateral radiographic knee osteoarthritis progression in women. Additionally, some studies have shown that disturbances in amino acid metabolism (e.g., Proline, tryptophan, cysteine, and glutamine) are frequently observed in patients with rheumatoid arthritis [39,40]. Given these findings, phenylalanine may play a pathological role rather than a beneficial one. However, it is essential to note that our study utilized samples from healthy individuals, which differ from the pathological samples examined in the literature, as mentioned earlier.

By contrast, various metabolite profiles displayed a curve shape that was opposite to the pharmacokinetics curve of Etodolac—for instance, prostaglandins, ganglioside-1, and phospholipids.

Etodolac, as a selective COX-2 inhibitor, suppresses the enzymatic conversion of arachidonic acid into pro-inflammatory prostanoids, including PGE1, PGE2, PGF1α, and prostacyclin (PGI2). The downregulation of these metabolites observed in our study directly reflects the expected pharmacodynamic effect of COX-2 inhibition, aligning with previous studies that demonstrate significant prostaglandin reduction following NSAID administration [19,41].

In addition, Ganglioside-1 (GM1) is a sialic-acid-containing glycosphingolipid that anchors to the cell surface, with its lipid chains embedded in the plasma membrane bilayer and its glycan portion exposed on the outer leaflet. It is highly enriched in the central and peripheral nervous system, where it serves as an essential component of neuronal cells and is critical for brain function [42]. Notably, excessive accumulation of GM1 is associated with the inherited neurodegenerative disorder named GM1 gangliosidosis [43].

We observed a decrease in GM1, a glycosphingolipid involved in membrane organization and inflammatory signaling. While the connection between COX-2 inhibition and sphingolipid metabolism is less direct, existing evidence suggests that NSAIDs can modulate lipid raft composition and influence sphingolipid-mediated pathways involved in cytokine signaling and immune cell activation [44,45]. The reduction in GM1 may reflect Etodolac’s secondary metabolic effects on membrane lipid composition and neuroimmune function, consistent with broader systemic shifts following inflammation suppression.

#### 3.1.2. Metabolic Alterations Specifically at the Cmax Time Point of Etodolac Administration

Our findings showed that, at the Cmax time point, Etodolac administration caused systemic metabolic alterations in metabolic pathways including arachidonic acid metabolism, sphingolipid metabolism, and biosynthesis of unsaturated fatty acids. Etodolac altered arachidonic acid metabolism, which is consistent with its known mechanism of action [46,47]. Mechanistically, arachidonic acid is considered the direct precursor of eicosanoids such as prostaglandins, leukotrienes, and epoxyeicosatrienoic acid obtained from three distinct enzymatic systems, namely the cyclooxygenase pathway, lipoxygenase pathway, and cytochrome P450 pathway [48]. Arachidonic acid (AA) is a 20-carbon-long-chain polyunsaturated fatty acid provided in two ways. AA comes from dietary polyunsaturated fatty acids or the conversion of cell-membrane-derived phospholipids through phospholipase A_2_ (PLA_2_) [49]. AA is converted to prostaglandin H2 (PGH2) through the action of COX-1 or COX-2 [50,51]. Since PGH2 is an unstable metabolite, it is converted into various derived prostaglandins, such as PGE2, PGI2, and PGD2, which are involved in pain and inflammation, blood vessel contractions, and adipogenesis, respectively. Previously, a comparison study illustrated that Etodolac has an inhibitory effect on the biosynthesis of PGE2 [52]. In our findings, we report that PGE2 was downregulated post-Etodolac administration, which is consistent with the literature.

Moreover, our findings revealed a consistent decrease in multiple prostaglandins following Etodolac administration, including PGE1, PGF1α, and prostacyclin (PGI2). This collective downregulation aligns with Etodolac’s known mechanism as a selective COX-2 inhibitor. Notably, PGI2, synthesized via COX-2 activity in endothelial cells and involved in vasodilation and inhibition of platelet aggregation, was also significantly reduced in our samples, further supporting the drug’s impact on prostaglandin biosynthesis. After Etodolac administration, there are contrasting alterations in the levels of different lipids and fatty acids. Our results indicate that the sphingolipid pathway was notably affected by Etodolac administration. Specifically, the level of GlcCer (d18:1/25:0), a glycosphingolipid involved in cellular signaling and membrane structure, was significantly reduced. Sphingolipids including sphingosine, ceramide, sphingosine-1-phosphate (S1P), and ceramide-1-phosphate. These are bioactive lipids involved in growth regulation, cell migration, adhesion, apoptosis, senescence, and inflammatory responses [53]. Sphingolipids have pro-inflammatory properties because they can induce the action of COX-2, leading to the production of prostaglandins to support their inflammatory roles [54]. In addition, studies have shown that sphingolipids can either inhibit or activate cytosolic phospholipase A2 alpha (cPLA_2_α), one of the 30 known members of the Phospholipase A2 (PLA_2_) enzyme family, resulting in the induction of COX-2 gene expression, modulating the levels of prostaglandins [49]. Thus, it is plausible that sphingolipids are directly or indirectly affected by Etodolac, supporting the decreases in the production of prostaglandins and other related metabolites as mechanisms that enhance Etodolac-mediated COX-2 inhibition. Further validation studies are required to explore this possibility, which could be utilized as a therapeutic approach in the treatment of inflammation and pain.

Moreover, phospholipids and their derivatives were also found to be impacted by Etodolac, such as phosphatidylglycerophosphate (PGP), PGP (i-12:0/i-12:0), and PGP (a-13:0/i-12:0), which is a type of phospholipid involved in maintaining membrane structure and metabolism, that were down- and upregulated, respectively.

In the pathway analysis results, the fatty acid elongation, fatty acid biosynthesis, and fatty acid degradation pathways were identified as being affected. These results are still unclear. Various activated fatty acids were elevated post-Etodolac administration, such as palmityl-CoA, 6-hydroxytetradecanedioyl-CoA, 3-oxotetradecanoyl-CoA, 3-oxooctadecanoyl-CoA, and (9E)-10-nitrooctadec-9-enoyl-CoA, while other fatty acids were downregulated, including isodocosanoyl-CoA and docosanoyl-CoA. Collectively, Etodolac impacted the metabolism of different lipids and fatty acids through various mechanisms to achieve its overall systemic effects.

Our study confirms the well-established effect of etodolac on the arachidonic acid pathway, specifically through the inhibition of cyclooxygenase enzymes (COX-1 and COX-2), resulting in altered levels of key prostaglandins and related metabolites. This direct impact on arachidonic metabolism is evidenced by the significant changes observed in metabolites such as prostaglandin E2 and related lipid mediators, which are central to inflammation and pain signaling.

Beyond the arachidonic acid pathway, our metabolomic profiling reveals etodolac’s broader influence on other metabolic pathways. Notably, we observed significant dysregulation in amino acid metabolism, as indicated by altered levels of metabolites such as D-phenylalanine and glutamylalanine. These changes suggest modulation of protein turnover and signaling molecules derived from amino acids, which may contribute to the drug’s overall pharmacological effects.

Additionally, alterations in lipid remodeling pathways were apparent, with changes in metabolites like lyso-phosphatidylinositol and CDP-diacylglycerols, reflecting impacts on membrane lipid composition and signaling. Changes in energy metabolism intermediates also hint at a systemic metabolic response to etodolac administration, potentially linked to its anti-inflammatory actions.

Together, these findings demonstrate that etodolac’s metabolic effects extend beyond its classical target pathway, highlighting the complexity of its biological impact. This expanded understanding may provide new insights into the mechanisms underlying both its therapeutic benefits and side effects.

## 4. Materials and Methods

### 4.1. Ethical Approval

This study was conducted in accordance with the Helsinki Declaration. All participants signed an informed consent form and were free to withdraw from the study at any time. The Institutional Review Board (IRB) at Jordan Center for Pharmaceutical Research (JCPR), Amman, Jordan, reviewed and approved the study (IRB-01-R04).

### 4.2. Participants’ Demographic and Clinical Data

The study’s inclusion criteria primarily emphasized selecting healthy individuals to evaluate the metabolic effects of Etodolac without the confounding influence of existing health comorbidities. The study was intentionally designed to include participants of all ages and both sexes. Because of cultural limitations and the requirement for participants to stay overnight in a restricted setting, only males were able to participate in the study. Moreover, the health-based screening criteria resulted in the final participant group being limited to individuals between the ages of 19 and 41 years.

Thirty healthy male participants (*n* = 30) were enrolled in and completed the study. Participants were aged between 19 and 41 years old (25 ± 6.8) and had a body mass index (BMI) between 18.8 and 29 kg/m^2^ (23.8 ± 3.29). The volunteers were hosted in a clinical facility 6 h before study inception. The clinical facility provided all the participants with the same meals and drinks during the study for 48 h.

Furthermore, hematological, biochemical, immunological, and urinalysis parameters were recorded for each subject. The data are presented as means ± SD, percentage, or as negative/positive.

### 4.3. Blood Sample Collection Pre- and Post-Etodolac Administration

Patients were given a single oral dose of an Etodolac^®^ 400 mg tablet (Taro Pharmaceutical Industries Ltd., Hawthorne, NY, USA). A total of 150 blood samples were collected from the 30 volunteers, with 5 samples obtained from each participant at defined time points: pre-dose, prior to maximum concentration (Cmax), at Cmax, post-Cmax, and 36 h after dosing. Plasma was separated from these samples for pharmacokinetic and metabolomic analyses.

### 4.4. Assessment of Etodolac Pharmacokinetics

A selective and sensitive validated analytical method was used for the quantitative determination of Etodolac. The concentration of Etodolac in the plasma samples was measured using reversed-phase high-performance liquid chromatography (Agilent Technologies, Santa Clara, CA, USA) coupled with an Applied Biosystems Qtrap mass spectrometer (Sciex, Framingham, MA, USA). Pharmacokinetic parameters (Cmax and AUC0-t) for Etodolac were determined from the concentration data using non-compartmental analysis version 8.6 (Phoenix WinNonlin). The overall pharmacokinetic curves (concentration vs. time) were plotted, where the area under the curve (AUC) included concentrations from 0 to 36 hrs post-Etodolac dose. The Cmax of Etodolac refers to the maximum plasma concentration of the drug, where AUC0-inf was considered as a secondary parameter.

### 4.5. Sample Preparation and Metabolite Extraction

One hundred fifty plasma samples were subjected to label-free untargeted metabolomics analysis using high-resolution liquid chromatography–mass spectrometry (LC-MS). Metabolites were extracted using a previously published protocol [55]. Plasma samples of 100 μL were mixed with 900 μL of extraction solvent (1:1, *v*/*v*) ACN in MeOH and then thoroughly mixed on a thermomixer (Eppendorf, Hamburg, Germany) at 600 rpm at room temperature (RT) for 1 h. Afterward, the samples were centrifuged (Eppendorf, Hamburg, Germany) at 16,000 rpm at 4 °C for 10 min. In total, 950 μL of the supernatant was transferred into a 1.5 mL polypropylene microcentrifuge tube and then dried in a centrifugal vacuum evaporator at room temperature (RT) (Christ, Hagen, North Rhine-Westphalia, Germany). The dried extract samples were reconstituted with 100 μL of mobile phase (1:1) (*v*/*v*) A:B (A: 0.1% formic acid in dH_2_O; B: 0.1% formic acid in 1:1 (*v*/*v*) ACN:MeOH) and introduced to the LC-MS system [55].

To validate the stability of the LC-MS system throughout the run, quality control (QC) samples were prepared by pooling equal amounts of the 150 plasma samples in a single vessel. QC samples were extracted using the same extraction protocol and analyzed following the routine protocol. The acceptance criteria required that all QC samples be distinctly separated from other study groups, clustered together, and have a Relative Standard Deviation (RSD%) of less than 40%.

### 4.6. LC-MS-Based Untargeted Metabolomics Analysis

Metabolic profiling was conducted using the Waters Acquity UPLC system combined with an Xevo G2-S QTOF mass spectrometer equipped with an ESI. The extracted metabolites were analyzed on an ACQUITY UPLC with an XSelect C18 (100 × 2.1 mm, 2.5 μm) column (Waters Ltd., Elstree, UK). Mobile phase A consisted of 0.1% formic acid in dH2O, while mobile phase B contained 0.1% formic acid in a 1:1 (*v*/*v*) mixture of ACN and MeOH. The metabolites underwent chromatographic separation via gradient elution as follows: 0–16 min at 95–5% A, 16–19 min at 5% A, 19–20 min at 5–95% A, and 20–22 min at 95–95% A at a flow rate of 300 µL/min. Mass spectra were recorded in both positive (ESI+) and negative (ESI−) electrospray ionization modes. The eluted metabolites were ionized at a source temperature of 150 °C, with the desolvation temperature set to 500 °C. The capillary voltage was 3.20 kV (ESI+) or 3 kV (ESI−), the cone voltage was 40 V, the desolvation gas flow was 800.0 L/h, and the cone gas flow was 50 L/h. The collision energy for the low and high functions was set to 0 and 10–50 V, respectively, in MSE mode. The mass spectrometer was calibrated using sodium formate in the 100–1200 Da range. The lock mass compound, leucine-enkephalin (an external reference to the ion *m*/*z* 556.2771 in (ESI+) and 554.2615 (ESI−)), was injected continuously, alternating between the sample and the reference every 45 and 60 s for ESI+ and ESI−, respectively, with a scan time of 0.5 s, a flow rate of 10 µL/min, a cone voltage of 30 V, and a collision energy of 4 V. Data were acquired in continuum mode using the Masslynx™ V4.1 workstation (Waters Inc., Milford, MA, USA) [56].

### 4.7. Statistical Analysis

The MS raw data were processed using Progenesis QI v.3.0 software from Waters (Waters Technologies, Milford, MA, USA). A standard pipeline was employed, which began with alignment based on the *m*/*z* value and retention time of the ion signals, followed by peak picking and signal filtering based on peak quality. This was then followed by multivariate statistical analysis conducted with MetaboAnalyst v. 5.0 (McGill University, Montreal, Canada) (http://www.metaboanalyst.ca, accessed on 20 January 2025) [57]. The imported dataset was median-normalized, Pareto-scaled, and log-transformed to retain its normal distribution. The normalized datasets were used to generate partial least squares discriminant analysis (PLS-DA) and orthogonal projections to latent structures discriminant analysis (OPLS-DA) models. The created OPLS-DA models were evaluated based on the model fit (R^2^Y) and predictive ability (Q^2^) values using a permutation validation of 1000 samples.

Univariate analysis was performed using Mass Profiler Professional software v15.0 (Agilent, Santa Clara, CA, USA). One-way analysis of variance (ANOVA) with Tukey’s post hoc analysis (*p* < 0.05) was conducted among time points. A fold change (FC) cut-off of 2 was applied between the two chosen time points (pre-dose and Cmax). Pathway analysis was conducted to identify significant metabolic changes. A similarity test based on Pearson correlation was executed by comparing the metabolic profile of Etodolac with those of other metabolites. The comparison was made based on the direction of the curve throughout the study, highlighting metabolites that were similar to or different from the Etodolac profile.

### 4.8. Metabolite Identification

In Progenesis QI v.3.0 software, key metabolite features within each dataset were selected and annotated for peak identification. The chemical structures of these metabolites were determined by acquiring their exact precursor masses, fragmentation patterns, and isotopic distributions. These data were then cross-referenced with several databases, including the Human Metabolome Database (HMDB), METLIN MS/MS (https://metlin.scripps.edu/landing_page.php?pgcontent=mainPage), accessed on 12 February 2025, MassBank, LipidMap, LipidBlast, and the Kyoto Encyclopedia of Genes and Genomes (KEGG), utilizing a 5 ppm mass window [58]. Exogenous compounds, such as drugs, food additives, and environmental chemicals, were excluded from the final list of metabolites.

## 5. Conclusions

**Figure 5:**
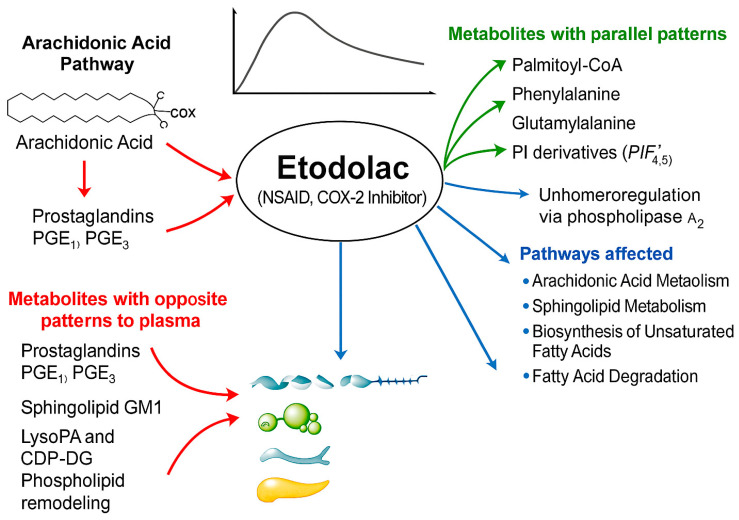
Systemic metabolic impact of Etodolac in healthy individuals.

This study focused on the systemic metabolic alterations associated with Etodolac administration in healthy individuals, providing insight into the drug’s mechanisms beyond its well-known action of inhibiting COX-2 and its involvement in broader metabolic regulation Figure 5. This study has its strengths, but also limitations. For the strengths of the study, a holistic, untargeted metabolomics approach enabled the discovery of novel metabolic changes. The use of healthy participants helped isolate the direct effects of Etodolac without interference from disease-related factors, with the potential to inspire future research into drug repurposing or combination therapies based on metabolic signatures. Importantly, the study revealed metabolic effects extending beyond arachidonic acid metabolism, including significant alterations in sphingolipid metabolism and fatty acid biosynthesis, offering deeper mechanistic insights into Etodolac’s systemic actions.

However, the study showed certain limitations that should be taken into consideration and avoided for future studies. The sample size was relatively small and only male participants were included, which may limit the statistical power and generalizability of the findings. Also, the study exclusively involved healthy individuals, which may not fully capture the drug’s impact in diseased states such as rheumatoid arthritis or chronic pain conditions. One notable limitation of this study is that approximately 80% of participants were smokers. Although all subjects were otherwise healthy and served as their own controls in a repeated-measures design, smoking is known to influence metabolic pathways, including those related to oxidative stress, lipid metabolism, and xenobiotic processing. Therefore, the potential confounding effect of smoking on baseline metabolite levels and drug response cannot be excluded.

Future studies should include patient populations with inflammatory or pain-related diseases, employ multi-omics integration, and use larger cohorts to validate and expand on these findings. Moreover, future studies should also consider stratifying participants by smoking status or including it as a covariate in the analysis to better isolate drug-specific metabolic effects. Ultimately, our study lays the groundwork for a deeper understanding of Etodolac’s systemic effects and may contribute to the development of improved therapeutic strategies and personalized treatment approaches in clinical practice.

This schematic diagram illustrates the metabolic pathways affected by Etodolac, a COX-2-selective nonsteroidal anti-inflammatory drug (NSAID). Central to the diagram is Etodolac, which inhibits cyclooxygenase-2 (COX-2), thereby reducing the conversion of arachidonic acid to pro-inflammatory prostaglandins (PGE_2_, PGE_1_α, PGI_2_). The figure categorizes endogenous metabolites into two groups based on their temporal profiles relative to Etodolac plasma concentration. The green arrows indicate metabolites that followed a parallel pattern to Etodolac, including palmitoyl-CoA, phenylalanine, glutamylalanine, and phosphoinositide (PI) derivatives, suggesting a coordinated biological response. The red arrows highlight metabolites that exhibited an opposite pattern, such as prostaglandins (PGE_1_, PGE_3_), sphingolipids (GM1, GlcCer), and phospholipids (LysoPA, CDP-DG), indicative of regulatory or compensatory processes. The blue arrows represent key metabolic pathways significantly affected by Etodolac as revealed by untargeted metabolomics: arachidonic acid metabolism, sphingolipid metabolism, biosynthesis of unsaturated fatty acids, and fatty acid degradation. Together, this diagram visually summarizes Etodolac’s systemic metabolic footprint, extending beyond its classical anti-inflammatory mechanism.

## Figures and Tables

**Table 1:** Demographic and clinical characteristics of participants.

**Demographic Data**	**Mean ± STD (*n* = 30)**	Normal Range *
Age (y)	25 ± 6.8	NA
Height (m)	1.73 ± 0.049	NA
Weight (Kg)	71 ± 9.7	NA
* Body mass index (BMI) (Weight/“Height^^2^”)	23.8 ± 3.29	NA
Smoker (%)	80%	NA
**Biochemical Data**	**Mean ± STD (*n* = 30)**	Normal Range *
Fasting blood sugar (mg/dL)	95.50 ± 8.577	70.00–115.00 mg/dL
Urea (mg/dL)	28.6 ± 7.72	10.0–50.0 mg/dL
Creatinine (mg/dL)	0.85 ± 0.146	0.60–1.30 mg/dL
Sodium (mmol/L)	143 ± 4.8	135–153 mmol/L
Potassium (mmol/L)	4.2 ± 0.39	3.50–5.30 mmol/L
Aspartate transaminase (SGOT) (U/L)	23 ± 3.4	Up to 42 U/L
Serum glutamate pyruvate transaminase (SGPT) (U/L)	18 ± 5.7	Up to 50 U/L
Alkaline phosphatase (ALP) (IU/L)	99 ± 22.2	Up to 40–150 U/L
Total bilirubin (mg/dL)	0.5 ± 0.25	Up to 1.40 mg/dL
**Hematological Data**	**Mean ± STD (*n* = 30)**	Normal Range *
Red blood cells (RBCs)	5.30 ± 0.383	4.20–6.10 10^12^/L
Hemoglobin	15.82 ± 0.846	14.00–18.00 G/DL
Hematocrit	46.62 ± 2.343	40.00–54.00%
Mean corpuscular volume (MCV)	88.20 ± 3.763	76.00–94.00 FL
Mean corpuscular hemoglobin (MCH)	29.90 ± 1.432	26.00–31.00 PG
Mean corpuscular hemoglobin concentration (MCHC)	33.90 ± 0.583	31.00–36.00 G/DL
White blood cells (WBCs)	7.75 ± 2.233	4.50–11.00 10^9^/L
Neutrophils	55.87 ± 6.056	45.00–75.00%
Lymphocytes	36.20 ± 5.610	25.00–40.00%
Monocytes	5.07 ± 1.617	0.00–7.00%
Eosinophils	2.33 ± 1.768	0.00–4.00%
Basophils	0.53 ± 0.507	0.00–1.00%
Platelets	229.43 ± 50.665	150.00–450.00 10^9^/L
Immunological Data	Mean ± STD (*n* = 30)	Normal Range *
Hepatitis B surface antigen (HBs Ag)	Negative	Negative < 1.00 S/CO
Hepatitis C virus antibody (HCV Ab)	Negative	Negative < 1.00 S/CO
Human immunodeficiency virus types I and II (HIV I and II)	Negative	Negative < 1.00 S/CO

* Body Mass Index (BMI) [underweight: less than 18.5; healthy weight: 18.5 to 24.9; overweight: 25 to 29.9; obese: 30 or higher].

**Table 2:** A summary of annotated endogenous metabolites showing significant changes post-etodolac administration. The metabolites are grouped by their pattern of regulation relative to etodolac: those exhibiting a similar pattern (increased or decreased concordantly with etodolac levels) and those showing an opposite pattern (inverse changes relative to etodolac).

Pattern	Annotated Metabolites	Reference
Similar pattern	CDP-DG(18:1)-O(12,13)/20:4), lysoPI(20:4/0:0), PIP(TXB2/22:4), PIP(22:5)/PGE2), palmitoyl-CoA, D-phenylalanine, 3-oxotetradecanoyl-CoA, 6-hydroxytetradecanedioyl-CoA, glutamylalanine, 10-nitrooctadec-9-enoyl-CoA, O-acetyl-ADP-ribose, 3-oxooctadecanoyl-CoA, PGP(a-13:0/i-12:0), 4-hydroxy-5-phenyltetrahydro-1,3-oxazin-2-one, CDP-glycerol	Figure 3A and Table S8
Opposite pattern	Glycineamideribotide, MG(0:0/TXB2/0:0), prostaglandin E2, Glc-Cer(d18:1/25:0), ganglioside GM1(18:1/12:0), prostaglandin E1, 15d PGD2, lysoPA(19:0/0:0), 8,9-DiHETrE, deoxycholic acid glycine conjugate, prostaglandin B-1, chenodeoxycholylproline, PGP(i-12:0/i-12:0), cGAMP(2′-5′), 14,15-DiHETrE, MG(18:4/0:0/0:0), prostaglandin B1, lysoPA(i-14:0/0:0), CL(8:0/8:0/11:0/18:2), CDP-DG(18:1/18:1-2OH(9,10)), PC(PGE1/P-18:1), PS(22:4/6 keto-PGF1alpha), MG(0:0/16:0/0:0), PC(22:5/20:5), Isodocosanoyl-CoA, PE(22:6-OH(4)/22:4), docosanoyl-CoA	Figure 3B and Table S9

## Data Availability

The data presented in this study are available on request from the corresponding author. The data are not publicly available due to privacy and ethical restrictions related to human sample handling and confidentiality agreements.

## Supplementary Materials

The following supporting information can be downloaded at: https://www.mdpi.com/article/10.3390/ph18081155/s1, Comprehensive screening data of study participants: demographic, hematological, biochemical, immunological, and urinalysis profiles; Supplementary Table S1: Demographic characteristics of study participants; Supplementary Table S2: Hematological parameters of study participants; Supplementary Table S3: Differential leucocyte counts of study participants; Supplementary Table S4: Biochemical screening results of study participants; Supplementary Table S5: Immunological screening results (HBsAg, HCV Ab, and HIV I/II); Supplementary Table S6: Urinalysis results including physical, chemical, and microscopic examination; Supplementary Table S7: Endogenous metabolites with significant changes post-dose compared to pre-dose, Supplementary Table S8: List of 17 significantly upregulated metabolites at Cmax compared to pre-dose, Supplementary Table S9: List of 27 significantly downregulated metabolites at Cmax compared to pre-dose.
