# Supplementary material for: Systemic Metabolic Alterations Induced by Etodolac in Healthy Individuals
Source: Pharmaceuticals (Basel). 2025 Aug 4;18(8):1155. doi: 10.3390/ph18081155 (PMC12388885; doi:10.3390/ph18081155)
Supplement: Supplementary file 1 [file pharmaceuticals-18-01155-s001.zip › Supplementary tables S1-S6.pdf]

Supplementary Table S1: The Demographic Data of Participants

Subject No	Subject Initial	Screening Date	Date of Birth	Sex	Age	Height (Meter)	Weight (Kg)	BMI	Smoker*/ Non-Smoker
**01**	MJRH041	14/02/2022	01/04/1998	Male	24	1.78	64	20.2	Yes
**02**	NKRA025	14/02/2022	17/02/2001	Male	21	1.80	61	18.8	Yes
**03**	MHMA001	14/02/2022	29/08/1998	Male	23	1.78	73	23.0	No
**04**	AMMS028	14/02/2022	06/06/2000	Male	22	1.72	62	21.0	Yes
**05**	MKAA029	14/02/2022	02/12/2002	Male	19	1.63	53	19.9	No
**06**	MSSA027	14/02/2022	05/05/2001	Male	21	1.73	63	21.0	Yes
**07**	AASA035	14/02/2022	19/06/1997	Male	25	1.69	65	22.8	Yes
**08**	AAMA026	14/02/2022	10/12/1998	Male	23	1.72	82	27.7	No
**09**	MOMY010	14/02/2022	18/05/2003	Male	19	1.82	67	20.2	No
**10**	AMAT014	14/02/2022	25/02/1987	Male	35	1.76	65	21.0	Yes
**11**	KRMA004	14/02/2022	4/04/1988	Male	34	1.78	87	27.5	Yes
**12**	IMAA008	14/02/2022	26/06/1992	Male	30	1.73	68	22.7	Yes
**13**	MSSA009	14/02/2022	24/10/1992	Male	29	1.78	75	23.7	Yes
**14**	HASK015	14/02/2022	20/05/2002	Male	20	1.76	67	21.6	No
**15**	MAAA036	14/02/2022	17/11/1996	Male	25	1.73	82	27.4	Yes
**16**	ZMSA034	14/02/2022	9/04/2002	Male	20	1.80	63	19.4	Yes
**17**	SHMA022	14/02/2022	31/08/2002	Male	19	1.81	90	27.5	No
**18**	AHHA031	14/02/2022	3/04/1995	Male	27	1.71	77	26.3	Yes
**19**	ZKMA020	14/02/2022	16/05/2003	Male	19	1.68	61	21.6	Yes
**20**	MNMO037	14/02/2022	6/11/1981	Male	40	1.70	85	29.4	Yes

*Smoker: not more than 3 cigarettes/ day during sampling days and 10 cigarettes / day during the wash out period.

Subject No	Subject Initial	Screening Date	Date of Birth	Sex	Age	Height (Meter)	Weight (Kg)	BMI	Smoker*/ Non Smoker
**21**	AAAO038	14/02/2022	15/02/1982	Male	40	1.72	83	28.1	Yes
**22**	MNMA043	14/02/2022	11/10/2001	Male	20	1.71	67	22.9	Yes
**23**	ONMA042	14/02/2022	12/08/2001	Male	21	1.73	81	27.1	Yes
**24**	AHMA017	14/02/2022	10/12/2002	Male	19	1.69	82	28.7	Yes
**25**	INAA030	14/02/2022	25/02/1981	Male	41	1.70	83	28.7	Yes
**26**	QTSA040	14/02/2022	04/09/2001	Male	20	1.65	66	24.2	Yes
**27**	AKHJ039	14/02/2022	08/05/2002	Male	20	1.73	62	20.7	Yes
**28**	OHMA019	14/02/2022	23/11/2000	Male	21	1.74	63	20.8	Yes
**29**	SSMA007	14/02/2022	22/09/1996	Male	25	1.71	70	23.9	Yes
**30**	QHMA016	14/02/2022	22/01/2001	Male	21	1.64	69	25.7	Yes
**Arithmetic Mean**					**25**	**1.73**	**71**	**23.8**	**Yes= 24/ No = 6**
**STD**					**6.8**	**0.049**	**9.7**	**3.29**	
**CV %**					**27.38**	**2.83**	**13.56**	**13.81**	
**Min**					**19**	**1.63**	**53**	**18.8**	
**Max**					**41**	**1.82**	**90**	**29**	

*Smoker: not more than 3 cigarettes/ day during sampling days and 10 cigarettes / day during the wash out period.

Supplementary Table S2: The Hematological Data of Participants

HEMATOLOGY ON SCREENING							
Subject No.	R.B.C 4.20 – 6.10 10^{12}/L	Hemoglobin 14.00- 18.00 G/DL	Hematocrit 40.00 – 54.00 %	M.C.V 76.00 – 94.00 FL	M.C.H 26.00 – 31.00 PG	M.C.H.C 31.00 – 36.00 G/DL	W.B.C. 4.50 – 11.00 10^{9}/L
**01**	5.45	16.64	48.60	89.20	30.50	34.20	7.13
**02**	5.44	16.05	47.10	86.60	29.50	34.10	8.58
**03**	5.16	15.20	45.00	87.30	29.10	33.40	8.08
**04**	6.03	16.83	50.10	83.10	27.90	33.60	10.76
**05**	5.71	15.05	45.10	79.00	26.40	33.40	4.72
**06**	5.05	15.98	46.80	92.70	31.60	34.10	5.00
**07**	5.40	15.93	47.20	87.40	29.50	33.80	10.67
**08**	5.75	15.81	46.90	81.60	27.50	33.70	4.96
**09**	5.31	16.17	48.80	91.90	30.50	33.10	9.95
**10**	4.91	16.07	47.60	97.00	32.70	33.80	6.59
**11**	5.78	17.06	49.40	85.50	29.50	34.50	4.48
**12**	5.32	15.10	46.00	86.50	28.40	32.80	10.98
**13**	5.44	16.42	47.60	87.50	30.20	34.50	7.07
**14**	5.91	16.63	49.30	83.60	28.10	33.70	6.09
**15**	5.87	18.10	51.80	88.20	30.80	34.90	9.61
**16**	4.86	15.51	45.70	94.10	31.90	33.90	8.22
**17**	5.31	16.28	47.70	89.90	30.70	34.14	8.59
**18**	4.75	14.73	43.20	90.90	31.00	34.10	5.09
**19**	4.84	14.96	44.20	91.40	30.90	33.80	6.71
**20**	5.02	15.40	45.70	91.00	30.70	33.70	10.75

HEMATOLOGY ON SCREENING							
**Subject No.**	**R.B.C** 4.20 – 6.10 10^{12}/L	**Hemoglobin** 14.00- 18.00 G/DL	**Hematocrit** 40.00 – 54.00 %	**M.C.V** 76.00 – 94.00 FL	**M.C.H** 26.00 – 31.00 PG	**M.C.H.C** 31.00 – 36.00 G/DL	**W.B.C.** 4.50 – 11.00 10^{9}/L
**21**	5.09	15.29	44.80	88.00	30.00	34.10	5.00
**22**	5.76	16.52	49.70	86.30	28.70	33.20	6.15
**23**	4.77	14.23	41.80	87.60	29.80	34.00	9.53
**24**	4.57	14.66	42.50	92.90	32.10	34.50	4.13
**25**	5.41	15.51	47.30	87.40	28.70	32.80	11.00
**26**	5.22	16.36	46.40	88.90	31.30	35.30	6.58
**27**	4.88	14.40	43.20	88.60	29.50	33.30	7.54
**28**	5.12	15.73	45.80	89.50	30.70	34.30	9.32
**29**	5.42	16.09	47.80	88.20	29.70	33.70	10.53
**30**	5.41	15.85	45.60	84.20	29.20	34.70	8.56
**Mean**	**5.30**	**15.82**	**46.62**	**88.20**	**29.90**	**33.90**	**7.75**
**STD**	**0.383**	**0.846**	**2.343**	**3.763**	**1.432**	**0.583**	**2.233**
**Min**	**4.57**	**14.23**	**41.80**	**79.00**	**26.40**	**32.80**	**4.13**
**Max**	**6.03**	**18.10**	**51.80**	**97.00**	**32.70**	**35.30**	**11.00**

Supplementary Table S3: The Differential Leukocytes Count Data of Participants

DIFFERENTIAL LEUCOCYTES COUNT ON SCREENING						
**Subject No.**	**Neutrophils** 45.00 – 75.00 %	**Lymphocytes** 25.00 – 40.00 %	**Monocytes** 0.00 – 7.00 %	**Eosinophils** 0.00 – 4.00 %	**Basophils** 0.00 – 1.00 %	**Platelets** 150.00 – 450.00 10^9/L
**01**	55.00	40.00	5.00	0.00	0.00	190.00
**02**	51.00	42.00	7.00	0.00	0.00	350.00
**03**	60.00	30.00	5.00	4.00	1.00	286.00
**04**	66.00	25.00	7.00	2.00	0.00	160.00
**05**	50.00	40.00	7.00	3.00	0.00	201.00
**06**	57.00	40.00	1.00	1.00	1.00	150.00
**07**	65.00	35.00	0.00	0.00	0.00	257.00
**08**	50.00	40.00	5.00	4.00	1.00	278.00
**09**	67.00	25.00	7.00	1.00	0.00	270.00
**10**	50.00	40.00	7.00	3.00	0.00	221.00
**11**	50.00	40.00	5.00	4.00	1.00	250.00
**12**	50.00	40.00	5.00	4.00	1.00	314.00
**13**	65.00	33.00	2.00	0.00	0.00	237.00
**14**	50.00	40.00	5.00	4.00	1.00	196.00
**15**	60.00	30.00	5.00	4.00	1.00	170.00
**16**	55.00	40.00	5.00	0.00	0.00	256.00
**17**	55.00	40.00	5.00	0.00	0.00	292.00
**18**	57.00	35.00	6.00	2.00	0.00	249.00
**19**	60.00	30.00	5.00	4.00	1.00	222.00
**20**	59.00	33.00	6.00	1.00	1.00	180.00

DIFFERENTIAL LEUCOCYTES COUNT ON SCREENING						
**Subject No.**	**Neutrophils** 45.00 – 75.00 %	**Lymphocytes** 25.00 – 40.00 %	**Monocytes** 0.00 – 7.00 %	**Eosinophils** 0.00 – 4.00 %	**Basophils** 0.00 – 1.00 %	**Platelets** 150.00 – 450.00 10^9/L
**21**	50.00	40.00	5.00	4.00	1.00	190.00
**22**	50.00	45.00	5.00	0.00	0.00	266.00
**23**	50.00	40.00	5.00	4.00	1.00	190.00
**24**	50.00	40.00	5.00	4.00	1.00	150.00
**25**	65.00	25.00	5.00	4.00	1.00	220.00
**26**	50.00	40.00	5.00	4.00	1.00	168.00
**27**	55.00	35.00	5.00	4.00	1.00	278.00
**28**	50.00	40.00	5.00	4.00	1.00	263.00
**29**	64.00	28.00	7.00	1.00	0.00	210.00
**30**	60.00	35.00	5.00	0.00	0.00	219.00
**Mean**	**55.87**	**36.20**	**5.07**	**2.33**	**0.53**	**229.43**
**STD**	**6.056**	**5.610**	**1.617**	**1.768**	**0.507**	**50.665**
**Min**	**50.00**	**25.00**	**0.00**	**0.00**	**0.00**	**150.00**
**Max**	**67.00**	**45.00**	**7.00**	**4.00**	**1.00**	**350.00**

Supplementary Table S4: The Biochemical Data of Participants

BICHEMISTRY ON SCREENING									
**Subject No.**	**Fasting Blood Sugar** 70.00 – 115.00 mg/dl	**Urea** 10.0 – 50.0 mg/dl	**Creatinine** 0.60 – 1.30 mg/dl	**Sodium** 135 – 153 mmol/L	**Potassium** 3.5 – 5.3 mmol/L	**SGOT** Up to 42 U/L	**SGPT** Up to 50 U/L	**ALP** Up to 150 U/L	**Bilirubin Total** Up to 1.4 mg/dl
**01**	97.00	26.9	0.87	150	3.7	24	18	96	0.5
**02**	96.00	32.3	0.80	141	4.5	24	13	114	0.6
**03**	96.00	28.1	0.71	139	4.1	27	25	67	0.3
**04**	97.00	27.5	0.60	138	4.1	24	19	80	0.4
**05**	100.00	23.9	0.66	139	4.2	28	17	145	0.8
**06**	97.00	26.0	0.74	141	4.2	22	11	111	0.6
**07**	88.00	32.7	0.70	137	3.9	18	12	101	0.4
**08**	89.00	41.3	0.97	146	5.0	21	13	79	1.1
**09**	104.00	18.8	0.92	143	4.4	21	11	84	0.7
**10**	94.00	37.4	0.69	142	4.2	23	16	68	0.5
**11**	108.00	28.2	0.92	144	4.9	23	28	98	0.3
**12**	81.00	42.7	1.10	137	3.7	19	19	84	0.2
**13**	89.00	44.4	0.75	142	4.0	25	19	86	0.3
**14**	95.00	15.1	0.85	139	4.1	26	19	90	0.4
**15**	82.00	18.4	1.00	140	4.2	18	19	96	0.5
**16**	88.00	24.8	0.78	152	4.4	24	18	134	0.5
**17**	100.00	30.0	0.86	137	3.5	33	36	73	0.6
**18**	101.00	20.0	0.93	140	4.2	17	13	95	0.3
**19**	90.00	46.7	0.99	144	4.3	21	14	112	0.3
**20**	109.00	20.7	0.81	149	4.3	23	19	112	0.3

BICHEMISTRY ON SCREENING									
**Subject No.**	**Fasting Blood Sugar** 70.00 – 115.00 mg/dl	**Urea** 10.0 – 50.0 mg/dl	**Creatinine** 0.60 – 1.30 mg/dl	**Sodium** 135 – 153 mmol/L	**Potassium** 3.5 – 5.3 mmol/L	**SGOT** Up to 42 U/L	**SGPT** Up to 50 U/L	**ALP** Up to 150 U/L	**Bilirubin Total** Up to 1.4 mg/dl
**21**	104.00	28.0	0.78	149	5.1	23	20	102	0.2
**22**	93.00	28.2	0.76	148	4.2	20	11	85	0.6
**23**	86.00	24.6	0.78	151	4.0	22	19	96	0.3
**24**	91.00	22.6	0.87	140	3.8	26	25	101	0.5
**25**	102.00	25.6	0.74	149	4.8	20	15	72	0.8
**26**	103.00	29.5	0.82	139	4.2	21	27	107	0.9
**27**	92.00	27.0	0.87	151	3.9	21	13	109	1.2
**28**	107.00	33.0	1.07	144	4.0	26	16	150	0.4
**29**	110.00	23.2	0.94	138	3.7	25	22	82	0.7
**30**	76.00	29.4	1.29	141	3.9	25	15	144	0.3
**Mean**	**95.50**	**28.6**	**0.85**	**143**	**4.2**	**23**	**18**	**99**	**0.5**
**STD**	**8.577**	**7.72**	**0.146**	**4.8**	**0.39**	**3.4**	**5.7**	**22.2**	**0.25**
**Min**	**76.00**	**15.1**	**0.60**	**137**	**3.5**	**17**	**11**	**67**	**0.2**
**Max**	**110.00**	**46.7**	**1.29**	**152**	**5.1**	**33**	**36**	**150**	**1.2**

Supplementary Table S5: The Immunological Data of Participants

IMMUNOLOGY ON SCREENING			
**Subject No.**	**HBs Ag** Negative < 1.00 S/CO	**HCV Ab** Negative < 1.00 S/CO	**HIV I and II** Negative < 1.00 S/CO
**01**	Negative	Negative	Negative
**02**	Negative	Negative	Negative
**03**	Negative	Negative	Negative
**04**	Negative	Negative	Negative
**05**	Negative	Negative	Negative
**06**	Negative	Negative	Negative
**07**	Negative	Negative	Negative
**08**	Negative	Negative	Negative
**09**	Negative	Negative	Negative
**10**	Negative	Negative	Negative
**11**	Negative	Negative	Negative
**12**	Negative	Negative	Negative
**13**	Negative	Negative	Negative
**14**	Negative	Negative	Negative
**15**	Negative	Negative	Negative
**16**	Negative	Negative	Negative
**17**	Negative	Negative	Negative
**18**	Negative	Negative	Negative
**19**	Negative	Negative	Negative
**20**	Negative	Negative	Negative
**21**	Negative	Negative	Negative
**22**	Negative	Negative	Negative
**23**	Negative	Negative	Negative
**24**	Negative	Negative	Negative
**25**	Negative	Negative	Negative
**26**	Negative	Negative	Negative
**27**	Negative	Negative	Negative
**28**	Negative	Negative	Negative
**29**	Negative	Negative	Negative
**30**	Negative	Negative	Negative

Supplementary Table S6: The Urine Analysis Data of Participants

URINANALYSIS ON SCREENING													
PHYSICAL AND CHEMICAL EXAMINATION												Microscopic Examination	
**Subject No.**	**Color** Yellow/ clear	**SP Gravity** 1.003 – 1.035	**pH** 4.5 – 8.0	**Nitrate** Negative	**Protein** Negative	**Ketones** Negative	**Glucose** Normal	**Bilirubin** Negative	**Urobilinogen** Normal	**Leucocytes /Epi** Negative	**Blood/Hb** Negative	**Leucocytes** Up to 10/ul	**Erythrocytes** Up to 3/ul
**01**	Yellow	1.020	5.0	-ve	-ve	-ve	Nor	-ve	Nor	-ve	-ve	8	2
**02**	Yellow	1.025	5.0	-ve	-ve	-ve	Nor	-ve	Nor	-ve	-ve	1	1
**03**	Yellow	1.025	5.0	-ve	-ve	-ve	Nor	-ve	Nor	-ve	-ve	3	1
**04**	Yellow	1.025	5.0	-ve	-ve	-ve	Nor	-ve	Nor	-ve	-ve	3	1
**05**	Yellow	1.020	5.0	-ve	-ve	-ve	Nor	-ve	Nor	-ve	-ve	2	1
**06**	Yellow	1.020	6.0	-ve	-ve	-ve	Nor	-ve	Nor	-ve	-ve	4	1
**07**	Yellow	1.020	6.0	-ve	-ve	-ve	Nor	-ve	Nor	-ve	-ve	5	1
**08**	Yellow	1.020	6.0	-ve	-ve	-ve	Nor	-ve	Nor	-ve	-ve	5	1
**09**	Yellow	1.025	5.0	-ve	-ve	-ve	Nor	-ve	Nor	-ve	-ve	1	1
**10**	Yellow	1.025	5.0	-ve	-ve	-ve	Nor	-ve	Nor	-ve	-ve	3	1
**11**	Yellow	1.020	6.0	-ve	-ve	-ve	Nor	-ve	Nor	-ve	-ve	4	1
**12**	Yellow	1.025	5.0	-ve	-ve	-ve	Nor	-ve	Nor	-ve	-ve	1	1
**13**	Yellow	1.020	5.0	-ve	-ve	-ve	Nor	-ve	Nor	-ve	-ve	2	1
**14**	Yellow	1.020	6.0	-ve	-ve	-ve	Nor	-ve	Nor	-ve	-ve	4	1
**15**	Yellow	1.020	6.0	-ve	-ve	-ve	Nor	-ve	Nor	-ve	-ve	4	1
**16**	Yellow	1.025	5.0	-ve	-ve	-ve	Nor	-ve	Nor	-ve	-ve	1	1
**17**	Yellow	1.020	6.0	-ve	-ve	-ve	Nor	-ve	Nor	-ve	-ve	4	1
**18**	Yellow	1.025	5.0	-ve	-ve	-ve	Nor	-ve	Nor	-ve	-ve	1	1
**19**	Yellow	1.020	5.0	-ve	-ve	-ve	Nor	-ve	Nor	-ve	-ve	8	2
**20**	Yellow	1.025	5.0	-ve	-ve	-ve	Nor	-ve	Nor	-ve	-ve	3	1
**21**	Yellow	1.020	5.0	-ve	-ve	-ve	Nor	-ve	Nor	-ve	-ve	2	1
**22**	Yellow	1.020	6.0	-ve	-ve	-ve	Nor	-ve	Nor	-ve	-ve	5	1
**23**	Yellow	1.025	5.0	-ve	-ve	-ve	Nor	-ve	Nor	-ve	-ve	1	1
**24**	Yellow	1.020	5.0	-ve	-ve	-ve	Nor	-ve	Nor	-ve	-ve	2	1
**25**	Yellow	1.020	5.0	-ve	-ve	-ve	Nor	-ve	Nor	-ve	-ve	8	2

URINANALYSIS ON SCREENING													
PHYSICAL AND CHEMICAL EXAMINATION												Microscopic Examination	
**Subject No.**	**Color** Yellow/ clear	**SP Gravity** 1.003 – 1.035	**pH** 4.5 – 8.0	**Nitrate** Negative	**Protein** Negative	**Ketones** Negative	**Glucose** Normal	**Bilirubin** Negative	**Urobilinogen** Normal	**Leucocytes /Epi** Negative	**Blood/Hb** Negative	**Leucocytes** Up to 10/ul	**Erythrocytes** Up to 3/ul
**26**	Yellow	1.020	5.0	-ve	-ve	-ve	Nor	-ve	Nor	-ve	-ve	2	1
**27**	Yellow	1.025	5.0	-ve	-ve	-ve	Nor	-ve	Nor	-ve	-ve	3	1
**28**	Yellow	1.020	6.0	-ve	-ve	-ve	Nor	-ve	Nor	-ve	-ve	5	1
**29**	Yellow	1.020	5.0	-ve	-ve	-ve	Nor	-ve	Nor	-ve	-ve	2	1
**30**	Yellow	1.025	5.0	-ve	-ve	-ve	Nor	-ve	Nor	-ve	-ve	3	1
